# The Next Step in the Treatment of Stroke

**DOI:** 10.3389/fneur.2020.582605

**Published:** 2021-01-22

**Authors:** Nathanael Matei, Justin Camara, John H. Zhang

**Affiliations:** ^1^Department of Ophthalmology, University of Southern California, Los Angeles, CA, United States; ^2^Department of Physiology and Pharmacology, Loma Linda University, Loma Linda, CA, United States; ^3^Department of Anesthesiology, Loma Linda University, Loma Linda, CA, United States; ^4^Department of Neurosurgery, Loma Linda University, Loma Linda, CA, United States

**Keywords:** stroke therapy, intravenous thrombolysis, mechanical thrombectomy, delayed recanalization, neuroprotection, synergistic cocktail

## Abstract

Although many patients do not receive reperfusion therapy because of delayed presentation and/or severity and location of infarct, new reperfusion approaches are expanding the window of intervention. Novel application of neuroprotective agents in combination with the latest methods of reperfusion provide a path to improved stroke intervention outcomes. We examine why neuroprotective agents have failed to translate to the clinic and provide suggestions for new approaches. New developments in recanalization therapy in combination with therapeutics evaluated in parallel animal models of disease will allow for novel, intra-arterial deployment of therapeutic agents over a vastly expanded therapeutic time window and with greater likelihood success. Although the field of neuronal, endothelial, and glial protective therapies has seen numerous large trials, the application of therapies in the context of newly developed reperfusion strategies is still in its infancy. Given modern imaging developments, evaluation of the penumbra will likely play a larger role in the evolving management of stroke. Increasingly more patients will be screened with neuroimaging to identify patients with adequate collateral blood supply allowing for delayed rescue of the penumbra. These patients will be ideal candidates for therapies such as reperfusion dependent therapeutic agents that pair optimally with cutting-edge reperfusion techniques.

## Introduction

### What Is Stroke

Stroke is the rapid development of disturbance of cerebral function attributed to the interruption of blood supply. Stroke is diagnosed by imaging evidence of infarct and associated clinical findings. Magnetic resonance imaging (MRI) is most sensitive to cerebral infarct using diffusion weighted techniques to compare the extent of the ischemic core with the extent of perfusion abnormality. Changes in diffusion weighted imaging are apparent in the hyperacute setting. Other modalities can diagnose cerebral infarct in the acute setting including computed tomographic (CT), CT angiogram/perfusion, and diagnostic cerebral angiogram. The two main types of stroke are ischemic and hemorrhagic, with incidences of 85 and 15%, respectively (1), accounting for 13.7 million new strokes each year (2). Ischemic stroke is currently the 5th leading cause of mortality in the United States, and 9.5 million first-time cases have been reported globally in 2016 (2–4). More than one-third of stroke-cost is due to lost productivity rather than actual treatment, pressing for the development of treatments that promote and assist recovery post-ictus (5).

Due to the plethora of causes for ischemic stroke, syndrome characterization occurs roughly by a rule of quarters: 25% cardioembolic, 25% thromboembolic, 25% lacunar, and 25% due to other causes (6). Of note, the majority of acute coronary syndromes result from a rupture or erosion of an atherosclerotic plaque, followed by *in situ* formation of a thrombus on the plaque, causing arterial obstruction (7). Often, ischemic stroke occurs from embolic arterial occlusion—either cardioembolic, caused by atrial fibrillation or valvular heart disease, or thromboembolic, from atherosclerotic disease in the extracranial cervical carotid or vertebral arteries (1). Cerebral blood flow through collateral vessels near the embolic arterial occlusion may help prevent total ischemia and ameliorate hypoxia-induced damage; however, collateral cerebral blood flow is inefficient in maintaining neuronal function and viability within the ischemic core (6). Under anaerobic conditions, complex metabolic events result in irreversible damage and neuronal death (8).

### Current Understanding of Stroke

**Figure 1:**
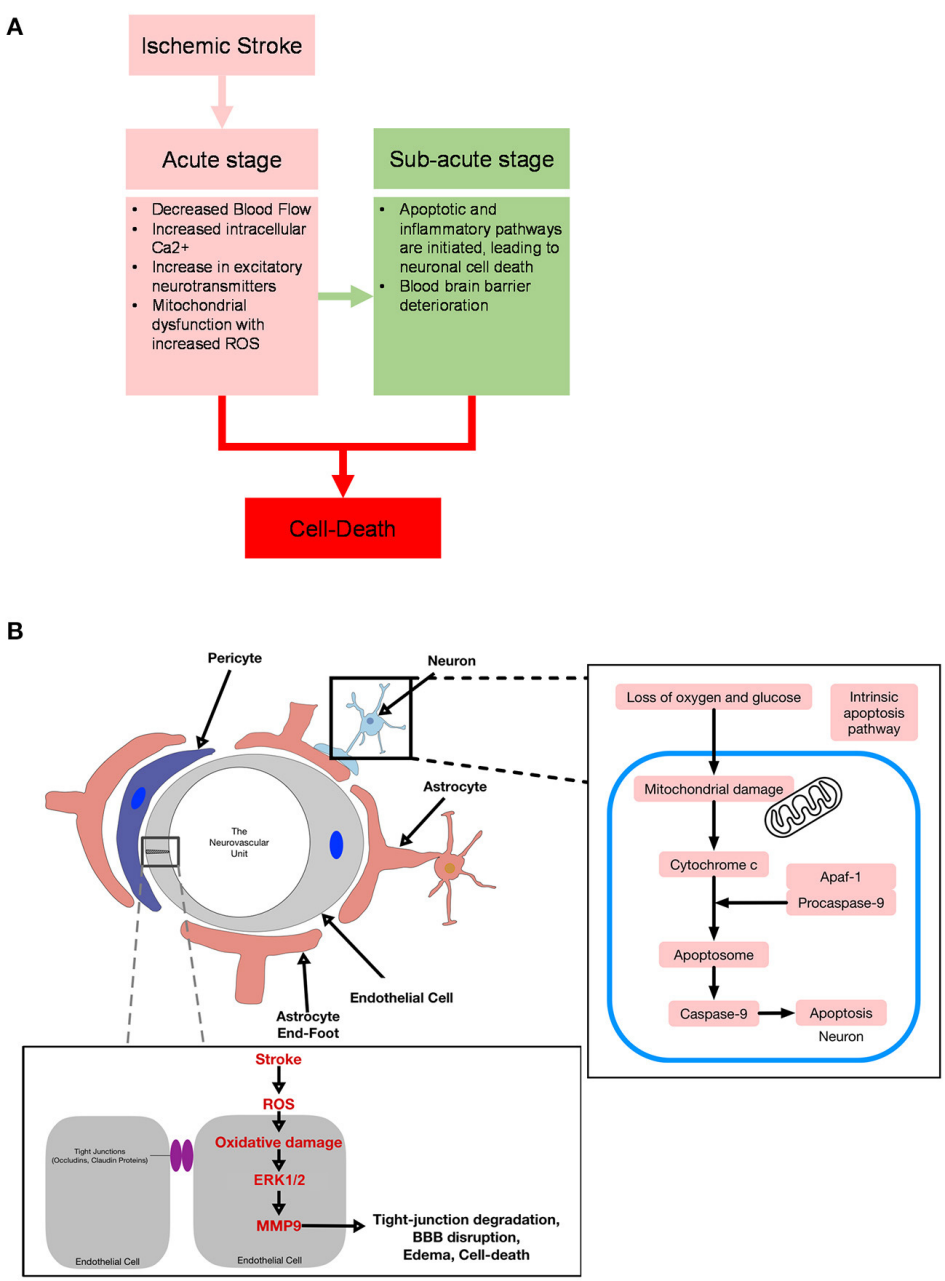
**(A)** Diagram for acute and sub-acute targets in stroke. **(B)** Diagram of ischemia effects on the neurovascular unit.

A foundational understanding of the etiology of stroke is important in the development of stroke therapeutics from first principles. In the acute stage of oxygen and glucose depletion in the brain, decreased blood flow disrupts ionic homeostasis and increases intracellular calcium stress responses. Intracellular calcium stress responses cause release of excitatory neurotransmitters and induce mitochondrial dysfunction, leading to generation of reactive oxygen species (ROS) (9). In the sub-acute stage, hours to days later, apoptotic and inflammatory pathways are then initiated, leading to neuronal cell death. Additionally, an increase in ROS and cytokines may lead to blood brain barrier deterioration, enabling protein and water to flood into the extracellular space, leading to vasogenic edema (10). The labyrinth of pathways observed in sub-acute stroke includes apoptosis, excitotoxicity, inflammation, and oxidative stress (Figure 1).

In recent pharmacological developments for stroke, attenuation of microcirculatory disturbances has relied on ablation of single factors in stroke pathogenesis with interventions including recombinant tissue plasminogen activator (rtPA), antioxidants, anti-intercellular adhesion molecule-1 (ICAM-1) antibodies, calcium-stabilizing agents, and anti-excitotoxic agents (10). Hitherto, rtPA continues to be the only FDA approved pharmacological intervention approved for acute ischemic stroke despite multiple clinical trials exploring alternative treatments (11). Although FDA approved, rtPA is not without side effects. Even when administered as indicated, rtPA increases the incidence of symptomatic hemorrhagic transformation (sICH) (12). Surveillance studies such as the Safe Implementation of Thrombolysis in Stroke-Monitoring Study (SITS-MOST) and Get With the Guidelines-Stroke (GWTG) demonstrated a small but significant increase in risk for sICH after rtPA administration despite slight variance in defining sICH (13, 14).

Large amounts of oxygen and glucose are needed to sustain normal brain metabolism. For example, to sustain the propagation of action potentials, 70% of the ATP supplied to the brain is used by Na+/K+ ATPase ion pumps located on the plasma membrane (15). After global ischemia, the depletion of oxygen and glucose results in the depletion of ATP within minutes, causing membrane depolarization, release of K+ into the extracellular space, entry of sodium into cells, and an increase in intracellular Calcium; if ATP is not restored, catabolic enzymes are activated and facilitate the necrosis of cells in the infarct (15). As necrosis establishes the ischemic core in the hypoperfused region, tissue at risk of cell-death around the core may survive for hours to days and may be salvaged with timely reperfusion and pharmacological treatment (16). The resulting blood brain barrier damage, inflammation, and apoptosis all contribute to the poor outcomes in ischemic stroke (17–19). Several recent clinical (Table 1) and basic science (Table 2) studies provide a basis for our perspective on the future of stroke management. Avoiding the pitfalls of previously failed therapies that we discuss extensively below, novel therapies and approaches may change the clinical management of stroke patients and provide a foundation for understanding and treatment of similar pathologies.

**Table 1:** Human studies.

**Author (year)**	**Stroke type**	**Treatment**	**Outcome**	**Significance**
Zhao et al. (20)	Acute stroke patients	Metformin combination treatment for 2 weeks	Improved neurological outcome (MMSE, NIHSS, and ADL scores) and reduced oxidative stress (increased GSH-PX and SOD)	*P* < 0.05
Lyden et al. (21)	Moderate to severe acute ischemic stroke patients	3K3-APC in combination with tPA, mechanical thrombectomy or both	Reduced IHC rate and total hemorrhage volume	*p* = 0.046
Vang et al. (22)	MCA LVO patients	Delayed recanalization >24 h	62% had improved CMCT	*P* = 0.005
Xavier et al. (23)	MCA LVO patients	Delayed recanalization at 15–29 days	86% achieved a mRS of 0–2	NA
Abou-Chebl et al. (24)	MCA, VBJ, ICA LVO patients	Delayed recanalization at 10–169 h	92% achieved a TIMI ≥2	NA
Tian et al. (25)	BA LVO patients	Recanalization at >2 days	~80% achieved a mRS score of 0–3	NA
Hill et al. (26)	ICA or MCAO LVO patients	Fingolimod plus alteplase (<6 h from last known well)	Reduced infarct volume, reduced blood lymphocyte counts, and improved mRS at day 90	P < 0.03
Chamorro et al. (27)	LVO patients	IV nerinetide plus thrombectomy +/– alteplase	mRS 0–2 at 90 days	Adjusted relative risk in patients receiving nerinetide but NOT alteplase: 1.18, 95% CI 1.01–1.38
Wu et al. (28)	MCAO LVO patients	Uric acid in combination to intravenous thrombolysis and mechanical thrombectomy	Improved mRS 0–2 at 90 days, reduced mortality (8–4%)	Functional outcome: 6.12, 95% CI 1.08–34.56 Mortality: 3.74, 95% CI 0.06–226.29
Wang et al. (29)	LVO patients	ISCF in combination to mechanical thrombectomy	No difference in mRS compared to controls	*P* = 0.2
Levy et al. (30)	Stroke patients	Stem cell treatment 4.2 ± 4.6 years post-stroke	Barthel Index improved by 10.8 ± 15.5 points at 12-months	*P* < 0.001
Muir et al. (31)	Stroke patients	Stem cell treatment 2–13 months post-stroke	three of twenty-three subjects demonstrated improved motor function at 6–12 months	NA
Kimberly et al. (32)	Stroke patients	IV glyburide	Improvements in midline shift, MMP-9 levels, NIHSS, and fewer deaths attributed to edema	*P* < 0.04

*ACA, anterior cerebral artery; BA, basal artery; CMCT, central motor conduction time; GSH-PX, glutathione peroxidase; ICA, internal carotid artery; ISCF, intraarterial selective cooling infusion; IV, intravenous; LVO, large vessel occlusion; MCAO, middle cerebral artery occlusion; MMP-9, matrix metalloproteinase 9; NIHSS, NIH Stroke Scale; SOD, superoxide dismutase; TIMI, thrombolysis in myocardial infarction; VBJ, vertebrobasilar junction; NA, not available*.

**Table 2:** Animal studies.

**Author (year)**	**Model**	**Treatment**	**Outcome**	**Significance**
McBride et al. (39)	Aged female mice (pMCAO) and hypertensive rats (MCAO)	3K3-APC plus tPA	Reduced infarct and improved neurological outcome	*P* < 0.01
Zheng et al. (143)	Rat MCAO	Delayed recanalization at 3, 7, and 14 days	3-day recanalization reduced the infarct volume. Recanalization in all groups improved neurological function	*P* < 0.05
Tang et al. (144)	Rat MCAO	Delayed recanalization at 3 days	Reduced infarct volume and improved neurological function	*P* < 0.05

*pMCAO, permanent middle cerebral artery occlusion; MCAO, middle cerebral artery occlusion*.

### Reasons for Failed Stroke Treatments

Scientific advances have birthed a plethora of neuroprotectants that were expected to have significant clinical efficacy. Specifically, a literature review of putative neuroprotectants with properly controlled *in vivo* and *in vitro* experiments using functional or histological endpoints and showing improvement in focal models, reported that 912 drugs (and well over 1,000 to date) have failed to achieve clinical success (33). One hypothesis for the failed translational success was suboptimal adherence to the Stroke Therapy Academic Industry Roundtable (STAIR) criteria, and subsequent studies will require improved rigor and analysis of animal data to ensure translational efficacy (33).

In the STAIR IX, expert opinions prioritized three main goals; one, given the development of endovascular therapy in treating acute stroke, current efforts have focused on enhancing access, procedural, and periprocedural aspects of this therapy; second, endovascular therapy remains underutilized because the lack of ubiquitous vascular imaging (CT angiography and magnetic resonance angiography) and inefficient triage of patients needed to facilitate treatment within the recommended window of intervention; and third, adjunct therapies, e.g., collateral flow stimulants, NA-1, Uric acid, Hypothermia, and Activated protein C, in combination with endovascular therapy may enhance the effect of reperfusion (34).

The consensus of STAIR X is that neuroprotective therapies for ischemic stroke have and continue to fail in clinical trials due to the complexity of stroke. First, stroke affects all the cells downstream to the occlusion, so a therapy targeting the neurovascular unit (Brain Cytoprotection) either by pleiotropic effects or improved reperfusion to the infarct core via collateral flow may be a better option compared to a single target approach that has been the primary focus of basic science research. Second, many of the failed clinical trials were designed as monotherapies instead of adjunctive therapies, which may be advantageously paired (pre or post) with current reperfusion approaches such as alteplase and thrombectomy devices. Third, failed monotherapeutic drugs should be reconsidered in adjunct to thrombectomy if they have shown significant improvement of outcomes in animal stroke models and demonstrated safety in phase II or III clinical trials (35).

Another possible factor in limited translational success might be failure of animal models to adequately represent the diversity of patient populations. Due to the small window for reperfusion, a majority of patients do not receive reperfusion. Although estimates vary and rates of thrombectomy/embolectomy are increasing over time, recanalization rates in patients receiving either medical or surgical intervention for large vessel occlusions range from 11 to 40% (36–38). Roughly 50% of patients either do not receive stroke intervention or fail to recanalize with therapy (39). Most animal models in stroke are reperfusion models, and the drugs developed in these models depend on the direct interaction of the drug in the infarct and penumbra zone. Shifting research to a permanent model of occlusion or developing ways to widen the reperfusion window (>24 h) would increase the chance of efficacy of new drugs and past drugs from bench to bedside.

A *post-hoc* subgroup analysis of the ESCAPE-NA1 trial demonstrated significantly improved outcomes in patients who received neuroprotectant nerinetide in combination with mechanical thrombectomy but without usual care alteplase (26). A drug-drug interaction of nerinetide and alteplase may have confounded or masked a neuroprotectant effect in the ESCAPE-NA1 trial (26). Aside from the ESCAPE-NA1 trial being the first large scale application of a neuroprotectant in the context of human ischemia-reperfusion, the study authors also propose that the study benefited from its design which closely paralleled the design and therapeutic timeline of preclinical animal studies (26, 40, 41). This logic can be applied in planning both preclinical and clinical studies. As reasonable, human trials should be designed to parallel the design of preclinical trials, and preclinical trials will ideally anticipate the eventual design of human trials. Failing to do so has likely contributed significantly to the dearth of successful neurotherapeutic trials. Other meaningful points of failure for past clinical trials include sex balancing, standardized criteria for image-based selection of patients (42–44), and variability in treatment protocol speed, especially in the context of poor collateral circulation (45–51).

The ESCAPE-NA1 trial failed to achieve significance in its primary outcome for the overall study population most likely due to proteolysis of nerinetide by the plasmin generated by alteplase. Future studies of neuroprotectants in human ischemia-reperfusion should be carefully evaluated for potential drug-drug interactions, and trials should be cognizant of this finding when selecting the optimal therapies to pair with improved mechanical/aspiration reperfusion therapies. In the context of patients also receiving alteplase, special care should be taken to ensure neuroprotectants are resilient against the enzymatic activity of activated plasmin (26).

## Stroke Pathophysiology: in Brief

### Cell-Death

With the loss of oxygen and glucose, a hypoxic state is created in the acute stage of cerebral infarction. If recanalization occurs, it may cause further stress on the brain, triggering neuronal cell apoptosis and loss of biological function (known as reperfusion injury) (52). A major contributor to outcome after stroke is the survival of neurons (53). Thus, a primary therapeutic target for the treatment of stroke has been protection of neurons (53, 54). In ischemic stroke, cell-death may occur via necrosis, which results from osmotic homeostatic imbalance and subsequent rupture of the plasma membrane (occurring within the first hours), or apoptosis, which occurs in a controlled manner through intrinsic and extrinsic pathways (occurring over several hours or days) (55). With necrosis occurring immediately after ischemic injury, apoptosis has been the focus of basic science research in efforts to prevent the recruitment of at-risk tissue, known as the penumbra, to the ischemic core. Of interest, literature has reported the activation of extrinsic and intrinsic pathways of caspase-mediated cell death in several forms of transient middle cerebral artery occlusion (MCAO) in adult rats (56). Stress-induced signaling events cause damage to DNA, cellular structures, and organelles—including cytoskeleton, mitotic microtubules, mitochondria, golgi, and sarcoplasmic reticulum—and leads to apoptosis (57).

Intrinsic pathways are activated when mitochondria are damaged, causing membrane depolarization and permeabilization, and subsequently releasing several proapoptotic factors from the mitochondrial space (58). Cyotochrome C is released and binds to cytosolic apaf-1 and procaspase 9 to form the apoptosome, resulting in the autoproteolytic activation of caspase 9 (57). Next, caspase 9 cleaves downstream effector caspases 3, 6, and 7, phenotypic markers of apoptosis (59, 60). In animal models of stroke, cleaved-caspase-3 is upregulated following stroke, and intervention through inhibition of caspase-3 reduces infarct size following transient MCAO [(56); Figure 1]. Thus, one reasonable approach to stroke intervention is to evaluate the efficacy of caspase-mediated cell death to provide translational therapeutic benefits following ischemic stroke.

In spontaneously hypertensive rats and aged female mice, 3K3A-APC, an altered version of activated protein c (APC) protease (3 sequential lysine residues replaced with 3 alanine residues) that has <10% of APC's anticoagulant activity, reduced infarct volume and improved behavior (29). Literature has shown that APC and 3K3A-APC activate their receptor, PAR1, expressed on brain endothelial cells, neurons and microglia, and promote neuronal survival via inhibition of downstream intrinsic and extrinsic apoptotic pathways (50). In a randomized, controlled, blinded phase II trial on the maximally tolerated dose (540 μg/kg) of 3K3A-APC in ischemic stroke patients (The RHAPSODY Trial), 3K3A-APC in addition to reperfusion (by tPA and/or mechanical thrombectomy) reduced intracranial hemorrhage rates (86–67%) and total hemorrhage volume (2.1 ± 5.8 to 0.8 ± 2.1 mL) compared to the placebo group; no difference in infarct volume was observed (21). Taken together, targeting the activation of cell-survival pathways, and thereby preventing the activation of apoptotic pathways, has been shown to improve outcomes following ischemic stroke.

Targeting cell-survival pathways, metformin treatment in 40 newly diagnosed acute stroke patients with type 2 diabetes mellitus resulted in improved neurological function and reduced oxidative stress (increasing the expression of glutathione peroxidase and superoxide dismutase) (20). To elucidate metformin's potential molecular mechanism in these patients, Zhao et al. treated oxygen-glucose deprived fetal rat hippocampal neurons with metformin and observed a reduction in the apoptotic rate via activation of the AMPK/pAMPK/mTOR/BAX/Bcl-2 pathway (20); many other basic science studies have also reported metformin treatment pre (61, 62) and post (63, 64) infarction to reduce infarct volume, neuronal apoptosis, and neurological deficits via pathways independent of its euglycemic effects.

### Inflammation

Secondary to necrosis/apoptosis, neuroinflammation occurs after cytokines are released by immune and CNS-resident cells (microglia, astrocytes, and neurons) (65). Clinically, cerebral edema resulting from inflammation is diagnosed with a non-contrast CT and includes symptoms such as changes in mental state and loss of consciousness, peaking at 72–96 h after stroke (66). Initially, ischemia activates microglia—resident immune cells that function as sensors and effectors—and then increases the infiltration of dendritic cells, macrophages, and lymphocytes (67). Inflammation may cause secondary damage to the initial lesion volume, e.g., increase in infarct growth and reperfusion injury, and although corticosteroids remain controversial, mannitol may be used as a treatment (66). Paradoxically, recanalization may increase infarct size and disseminate injury, an event known as ischemia-reperfusion injury (68–70). Although the mechanism of reperfusion injury remains incompletely characterized, apoptosis and necrosis activation of the inflammatory system have been implicated in ischemia-reperfusion injury (71). Therefore, inflammation must be monitored and accounted for in recanalized and non-recanalized patients.

To date, inflammatory treatments are targeting immunosuppression via fingolimod (pilot clinical trials) and inhibition of early platelet tethering and/or activation via glycoproteins. In a pilot trial, treatment with fingolimod (FTY720), an immunosuppressive drug used in multiple sclerosis that inhibits T cell activation via downregulation of the sphingosine 1 phosphate receptor, reduced infarct volumes and increased neurological outcomes compared to non-treated patients (72). In addition, T cell synergistic interactions with platelets are significant in ischemic-reperfusion injuries. For example, glycoprotein receptors expressed on platelets may bind to von Willebrand factor (VWF), facilitating adhesion/tethering to injured vessels (73). Once activated, glycoprotein receptors initiate procoagulant and pro-inflammatory pathways (73). In mouse models, the inhibition of the VWF-binding site on the glycoprotein IB-IX-V complex prevented the adhesion/tethering of platelets to damaged endothelial cells and reduced ischemic-reperfusion injury after middle cerebral artery reperfusion (50, 74). However, other reports have shown that the inhibition of platelet glycoproteins, such as IIb/IIIa, did not improve outcome compared to controls but activated pro-inflammatory pathways, suggesting a complex thrombo-inflammatory relationship in ischemic injury (50, 75). In a randomized, open-label, blinded endpoint clinical trial on patients with internal carotid artery or middle cerebral artery occlusions, acute combinational treatment (<6 h from infarct onset) with fingolimod and alteplase (manufactured tPA) reduced infarct volume and lymphocyte counts and improved neurological function at 3-months compared to alteplase treated patients (25). Thus, fingolimod enhanced the efficacy of alteplase administration in acute ischemic stroke patients. Given the heterogeneity of pathways involved in infarct growth and reperfusion injury, further research is needed to better understand pathway intersections to increase the efficacy of future treatments.

### Disruption of the Neurovascular Unit

Blood vessels carry blood from the heart to the tissue and are composed of arteries and arterioles (carry blood to tissue), the capillary bed (facilitate gas and nutrient exchange), and venules and veins (drain blood from tissue to the heart) (76). The blood-brain barrier (BBB) is the microvasculature barrier that regulates movement of molecules, ions, and cells into and from the central nervous system. The barrier is composed of two main cell types: endothelial cells that form the cell wall, and mural cells that sit on the abluminal surface of the endothelial layer (77). In acute ischemic stroke, BBB degeneration results from the disruption of tight junctions, vessel regression, brain hypoperfusion, and inflammatory responses.

BBB degeneration leads to pathological processes such as hemorrhagic transformation and/or edema, both of which exacerbate brain injury (78). MRI and CT imaging may be used to evaluate BBB disruption by measuring the extravasation of intravenously administered contrast material, and this dynamic contrast-enhanced MRI or CT may be combined with a pharmacokinetic model to quantify and spatially map BBB disruption (79). Assessment of BBB disruption may be deployed as a prognostic tool to predict risk of hemorrhagic transformation. To date, many etiological pathways have been suggested in ischemia induced BBB disruption, but additional research is needed to understand the exact molecular processes.

Ischemic insult may cause an increase in the swelling of the brain due to an influx of water content, a condition known as cerebral edema. The increased cerebral edema may lead to increased intracranial pressure, decreased cerebral blood flow, and even death through herniation (80). Comparing the types of edema, cytotoxic edema is apparent on diffusion weighted imaging, whereas vasogenic edema can contribute to significant mass effect, necessitating decompression (81). Cytotoxic edema occurs in up to 10% of patients with large infarcts with reported mortality rates of up to 80% (82). To date, there are two clinical approaches used to reduce cytotoxic edema: intravascular use of hyperosmolar solutions (such as Mannitol or hypertonic saline) and decompressive craniectomy. In MCA occlusion, cytotoxic edema can start within 30 min and even persist for up to 24 h after reperfusion. Specifically, given that astrocytes are involved in the clearance of K^+^ and glutamate, and that astrocytic but not neuronal Na-K-Cl co-transporter is upregulated after ischemia, astrocytes are more susceptible to water inflow (swelling) than neurons (83, 84). When the plasma membrane channels/pumps can no longer maintain cellular homeostasis, ischemic conditions activate oncosis pathways—a cellular death process that leads to necrosis with karyolysis (85, 86). In gray matter, ischemia results in the failure of energy dependent sodium/potassium membrane pumps and in the accumulation of intracellular Na^+^, thereby drawing chloride and water along their osmotic gradients, and resulting in cellular swelling (81). As intracellular fluid volumes increase, extracellular space decreases manifesting radiographically as restricted diffusion on MRI, demonstrating hyperintense signal on diffusion weighted imaging (DWI) and hypointense signal on ADC sequences (87).

The neurovascular unit compromises vascular cells (endothelial cells, mural cells such as pericytes of capillaries, venules, and precapillary arterioles), vascular smooth muscle cells, glial cells (astrocytes, microglia, and oligodendrocytes), and neurons (88). The prototypical function of the neurovascular unit is the coupling between neural activity and CBF. However, in ischemic stroke, BBB permeability increases due to the disruption of tight junctions via protein modification, translocation, and degradation, and the neurovascular unit is compromised as irreversible damage ensues (89). In detail, post-translational modification of tight junctions in response to ischemia may activate vascular endothelial growth factor, Rho/ROCK, and cyclic AMP/PKA, causing phosphorylation of tight junction proteins (occludin, claudin-5, and ZO-1) and thus increasing BBB permeability (89). Studies have predominantly focused on modulating the phosphorylation of tight junction proteins, but these proteins may also be regulated via methylation, glycosylation, acetylation, and/or palmitoylation, processes that could all play a role in BBB disruption (90). Furthermore, literature has shown that ischemia can activate PAR1 receptors, activating PKC-Akt and PKC-ERK1/2 pathways, and thereby increasing the release of matrix metalloproteinase-9 (MMP-9); MMP-9 degrades tight junction proteins such as occludin and claudin-5 (91). During ischemia, as ATP supplies are depleted and cellular stress increases, many of the cells in the neurovascular unit become compromised and die. For instance, endothelial cells have been reported to die acutely, sub-acutely, and chronically after infarct onset via activation of various pathways, e.g., lysosome-dependent, necroptosis, autophagy, and apoptosis, all of which may be potential targets for treatment (92). Along these lines, APC analogs, such as 3K3A-APC, have been reported to inhibit endothelial apoptosis through PAR1 and PAR3 receptors, stabilizing the endothelial cytoskeleton, and preserving the BBB (50, 93–95).

## Therapeutic Strategies

### Neuroprotection and Recanalization: Complementary Targets

The proximal aim of stroke therapy is to restore blood flow to the area of the infarction to reverse and minimize damage from oxygen and nutrient deprivation (96). In the acute phase, patients are given IV rtPA therapy or screened for mechanical thrombectomy. Research has established an extended therapeutic window in patients who meet standard clinical criteria and who have perfusion imaging evidence of salvageable ischemic penumbra 4.5–9 h after onset (or within 9 h of the midpoint of sleep in those with stroke recognized upon awakening) (97, 98). Patients with unknown onset stroke also benefit from thrombolysis if MRI demonstrates a diffusion lesion without changes on the FLAIR sequence which indicates that onset is likely to have been within the last 4.5 h (99). Further research is accumulating to extend the therapeutic window in patients who meet certain clinical and imaging criteria. Patients with sufficient penumbra beyond the initial ictus who present outside the traditional therapeutic window can still benefit from delayed treatment (24, 100, 101).

A secondary and complementary aim of stroke therapy is to use pharmacological agents to attenuate mechanistically deleterious stroke pathways that are activated by ischemia and reperfusion (96). Although mechanical and pharmacological recanalization resolve the proximal cause of brain injury, they also directly increase risk of hemorrhagic transformation, especially rtPA. Restoration of blood flow is accompanied by an increase in circulating inflammatory cells. Furthermore, full recanalization is often impossible due to surgical limitations or limited rtPA and plasminogen contact with the clot surface (<1% chance of recanalization when the clot exceeds 8 mm) (96); thus, to increase translational success of rtPA, endovascular therapy, and neuroprotective agents, a synergistic cocktail is most promising to overcome the barriers to effective treatment.

**Figure 2:**
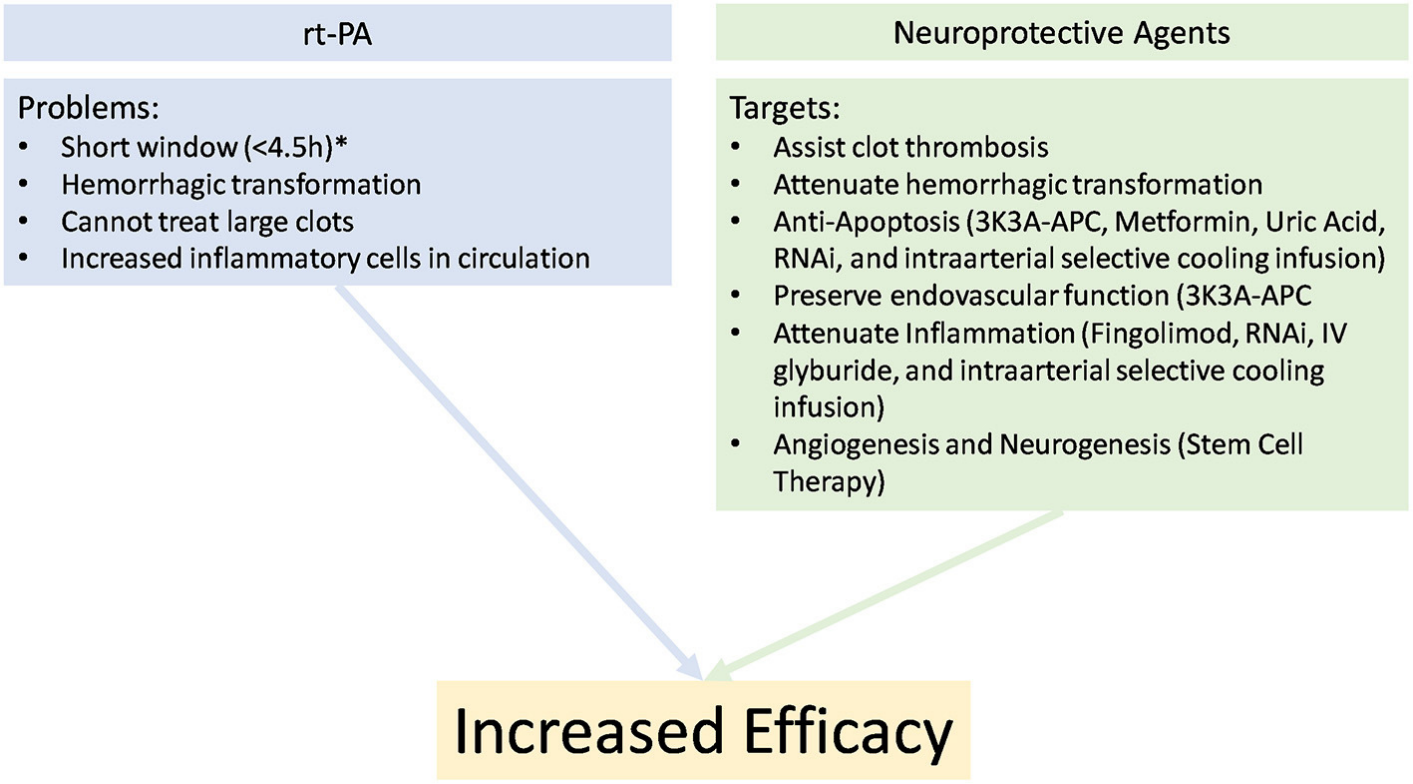
Potential explanations for poor clinical outcomes with rTPA alone and the beneficial properties of adding a synergistic cocktail for the treatment in stroke. *Imaging selection may allow later treatment.

Current evidence supports endovascular thrombectomy in patients appropriately selected with advanced clinical image, including patients who have already received rtPA for thrombolysis (100, 101). Patients are receiving intervention for stroke more frequently and longer after the onset of stroke (69, 97–99, 102), often beyond 6-h from onset, opening a new population of patients who will likely benefit from careful application of neuroprotective agents to target cell death, angiogenesis, and neurogenesis to reverse deleterious consequences in the penumbra (Figure 2). The ESCAPE-NA1 trial of nerinetide is the first large trial of any neuroprotective agent in the setting of human ischemia-reperfusion (26), emphasizing that although the field of neuronal, endothelial, and glial protective therapies has seen numerous large trials, the application of therapies in the context of newly developed reperfusion strategies is still in its infancy.

Uric Acid is a product of the catabolism of purine nucleotides and contributes up to 60% of the plasma antioxidant activity: scavenging hydroxyl radicals, superoxide anions, hydrogen peroxide, and peroxynitrite (103). In MCAO mouse models, uric acid treatment reduced infarct volume, ROS production, and neurological deficits (104, 105). In addition to antioxidant activity, studies suggest that the therapeutic potential of uric acid is predominately facilitated via effects on the vasculature. For example, in a rat MCAO model, uric acid treatment reduced MCA wall thickening and increased lumen expansion (106). Mechanistically, uric acid treatment increases the expression Kruppel-like factor 2 and reduces the expression of VEGF-A, thereby maintaining BBB integrity (103). In the URICO-ICTUS trial, a multicenter, randomized, double blind, phase 2b/3 trial, patients that received uric acid in combination with alteplase (<4.5 h) did not improve outcomes at 90 days, but in a sensitivity analyses, a borderline significance was seen in patients with uric acid treatment who experienced an ordinal shift in mRS, decreasing their residual disability by a median of 1 point on the mRS compared to the placebo group (107). Moreover, in a URICO-ICTUS trial subgroup analysis (45 out of 411 patients), uric acid in combination with intravenous thrombolysis (<4.5 h) followed by thrombectomy (*N* = 24) within 8 h after stroke improved functional outcomes compared to the placebo group (*N* = 21) (27). Taken together, therapies targeting BBB disruption may provide added protection in the mitigation of ischemia/reperfusion injury.

Recent developments in gene therapy such as RNA interference (RNAi), which are used as a natural defense against exogenous genes, can bind to specific mRNA to interfere with expression, thereby reducing the expression of upstream genes (108). Even though no RNAi treatment is FDA approved for stroke, animal studies have shown efficacy in siRNA treatment in brain hemorrhage, brain ischemia, and traumatic brain injury models (109). Specifically, recent *in vivo* studies have reported improved outcomes following ischemic stroke via inhibition of the following proteins involved in apoptosis, coagulation cascade, hypoxia induced cascade, and neuroinflammation: Caspase-3 (110), Beclin1 (111), Ask1 (112), PAR1 (113), HIF1alpha (114), GPR17 (115), and HMGB1 (116). Thus, siRNA can be applied to many pathophysiological pathways and may prove versatile in treating ischemic stroke. However, limitations persist in clinical delivery because the BBB excludes lipid-insoluble compounds and macromolecules (109).

Another approach to stroke treatment includes stem cell therapies in the subacute or chronic stages that target inflammation, neural plasticity, neovascularization, and growth factors in response to ischemic injury. In a single-arm, phase I clinical trial, intravenous administration of autologous bone marrow mononuclear cells (10 million cells per kilogram) within 24–72 h of stroke onset resulted in a reduction of 1 point in median day-90 mRS compared to the non-treated control group, wherein no severe adverse events were observed (117). In a phase 2 randomized trial (RECOVER-Stroke), internal carotid artery infusion of autologous bone marrow-derived ALD-401 cells 2-days following a bone marrow harvest at 11–17 days post-stroke resulted in no detectable improvement in mRS, Barthel Index, NIHSS, and EZ-5D scores (*N* = 48) (118).

In an open-label, single-arm, multicenter study (PISCES-2) investigating neurological function following intra-arterial injection of CTX0E03 cells (human neural stem cell line) 2–13 months after stroke, one of twenty-three and three of twenty-three subjects demonstrated improved motor function at 3 and 6–12 months, respectively, while no deleterious outcomes occurred related to the stem-cell therapy (31). In a Phase I/II study, intravenous allogeneic mesenchymal stem cells were administered at an average of 4.2 ± 4.6 years following stroke (*N* = 36). Patients enrolled in the trial demonstrated improved behavioral outcomes over the 12-months of follow-up, e.g., Barthel Index scores improved from a baseline of 11–27% at 6-months and to 35% at 12-months (*P* < 0.002) (30). Taken together, stem cell therapies are feasible and merit further investigation in future randomized, controlled trials for both subacute and chronic stroke patients.

## Opportunities for Advancement of the Field

In 2015, several landmark clinical trials comparing the efficacy pharmacological and mechanical thrombectomy for treatment of large vessel ischemic stroke were published (50). These studies support endovascular thrombectomy as the standard of care for large vessel stroke meeting clinical and imaging criteria with or without rtPA (119–121). Given the results of these studies, networks of care will continue optimization to maximize the fraction of patients receiving pharmacological thrombolysis, and when available, endovascular thrombectomy. Furthermore, recent trials have extended the eligible time window for reperfusion to 16 h and beyond given appropriate magnetic resonance imaging selection criteria (69, 122). These findings have significant translational relevance to findings in animal models of stroke. Neuroprotective treatments with limited efficacy in permanent occlusion, but significant improvement in infarction volumes and neurobehavioral outcomes after reperfusion would be strong candidates (123). These neuroprotective agents will likely perform well in combination with pharmacological thrombolysis or mechanical thrombectomy. Increasingly, more patients will be screened with early magnetic resonance imaging to identify patients with adequate collateral blood supply allowing for delayed rescue of the penumbra (124). These patients will be ideal candidates for therapies that pair optimally with reperfusion of viable parenchyma (94, 125, 126). The beneficial application of neuroprotective agents is not strictly limited to patients with excellent collaterals, but rather, these patients may more frequently be the target of pharmacological and mechanical attempts to rescue the penumbra, and thus are more likely to be in a situation to receive adjunctive therapies. Patients with moderate-to-large ischemic cores may also benefit from select neuroprotective agents despite limited bioavailability within the ischemic core. Small ischemic cores with adequate collateral supply will allow for optimal bioavailability of neuroprotectants, but the clinical efficacy of treatments may be small compared to reperfusion alone secondary to the mild nature of the disease. Conversely, moderate-to-large infarcts, which are often accompanied by poor collateral circulation to the penumbra, may achieve lesser bioavailability of neuroprotectants at the target site, but clinically may demonstrate improvement due to the greater initial severity of disease.

Standardized and widespread acceptance of newly developed stent retriever and/or direct aspiration catheters from manufacturers will increase the fraction of stroke patients achieving timely reperfusion (127–131). Although these devices have been utilized at some medical centers for several years, they are still at a relatively nascent stage of development. Finesse aspects of device design, including application in combination with or without balloon occlusion, are areas of ongoing research (132). This new, larger population of patients receiving timely reperfusion is distinct from previous experimental populations studied before the onset of skilled neuro-interventional providers with the latest tool set. These patients form a promising cohort for application of new and previously failed therapeutics.

Building on the foundation of recently established interventional techniques, we postulate several unique therapeutic applications that previously would not have been feasible. For instance, balloon occlusion techniques, when applied in combination with mechanical thrombectomy or direct aspiration, may transiently improve local bioavailability of intra-arterial therapies, delaying washout and systemic dilution, which may be especially efficacious in the context of blood brain barrier and endothelial-directed therapeutics. Theoretically, this could allow for much higher efficacy of therapeutics that would otherwise be limited in their systemic dosing or decreased in efficacy once normal, high-volume arterial perfusion is re-established. Similarly, intra-arterial administration of therapeutics at the moment of stent retriever deployment via a guide-catheter would ensure delivery of protective therapeutics to the at-risk vascular territory just before and during the moment of reperfusion.

Given that the DEFUSE (69) and DAWN (102) trials have extended recanalization in certain patients up to 24 h from infarct, reports are growing of delayed recanalization (>24 h) with favorable outcome in a variety of stroke subtypes. In MCA occlusions, recanalization at >24 h (22–24, 133, 134) and up to 60 days (135) since last known well has demonstrated improved outcomes. In basilar artery occlusions, reperfusion at 36 h (136), 50 h (137), and >2 days (138) resulted in fully restored neurologic function, complete functional recovery, and 77% mRS of 0–3, respectively. In internal carotid artery occlusions, recanalization from 1 month up to 27 months resulted in favorable outcomes, with some patients achieving full recovery (139–142). In basic science research, there is a limited number of studies that have investigated recanalization beyond 24 h. In MCAO rat models, investigators have shown delayed recanalization to improve neurological function as well as even reduce infarct volume when administered at 3-, 7-, and 14-days following stroke onset compared to permanent MCAO groups (39, 143, 144).

Clinical research has established the principle of “time is brain,” meaning that time delay before intervention is related to the loss of brain tissue (145). This principle emphasizes the need for early intervention in stroke, especially in patients with poor collateral blood supply. In practice, this requires that the planned intervention be feasible and easily deployed in the field. Although further research is needed to understand the pharmacokinetics and develop optimum dosing schedules, many neuroprotective agents would theoretically allow rapid deployment following positive findings on computed tomography screening or even administration by emergency response personnel in cases of high pre-test probability of ischemic stroke.

The concept of “time is brain” has been debated, but expert opinion holds that “time is brain” applies most directly to parenchyma lacking sufficient collaterals such that ischemia is primarily a watershed event without opportunity for rescue or subsistence level of perfusion supplied by collateral vessels. In real-world application, collateral circulation varies among individuals and directly affects the course of stroke injury (146).

Taken together, recent developments provide a wide frontier for advancement of the field. Improved imaging technology and interpretation provide the opportunity to test adjunctive therapies selected to pair optimally with reperfusion or collateral status. The logistics of ensuring maximum implementation of new state-of-the-art techniques and widespread, high-quality training of interventionalists with the latest equipment will be an ongoing opportunity for improvement in overall outcomes. Specific devices and techniques will be matched with rationally selected adjunctive therapies. The relationship of time and stroke intervention will continue to evolve from first responders to delayed treatment.

### Limitations and Potential Solutions

Because ~50% of withheld recanalization therapy is attributed to prehospital delays (147), extending the window for reperfusion and increasing efficacy of delayed recanalization penumbra need to be the focus of future studies. First, given the potential for immediate impact, trials should begin incorporating synergistic neuroprotective cocktails in combination with reperfusion therapy. Recently, in patients with internal artery or middle cerebral artery occlusions, combined treatment, fingolimod (an antagonist to Sphingosine receptors) and alteplase (manufactured tPA), reduced infarct volume and improved reperfusion and outcome compared to the alteplase treated group (25). Similarly, additional clinical trials are investigating neurological outcome in patients with proximal large vessel occlusions that undergo mechanical thrombectomies in conjunction with fingolimod (148). Along these lines, treatments targeting apoptosis, inflammation, edema, and BBB disruption may serve to be advantageous in combination with reperfusion.

The most significant contraindications to delayed recanalization are hemorrhagic transformation (HT) and mortality. Molina et al. have reported acute recanalization (<6 h) to result in HT in ~20% of patients compared to ~50% of patients in delayed recanalized groups (<24 h) (149). Because of the potential risks, more research is needed to investigate HT following cocktailed treatments targeting BBB integrity or even at reperfusion intervention beyond 24 h. In a clinical study evaluating mortality in patients >80 years of age, although not statistically significant due to low power, higher numbers of mortality resulted from reperfusion beyond 8 h from stroke onset (*P* = 0.055; *N* = 96) (150). Small study populations are susceptible to the effects of small sample sizes. Thus, additional studies are needed with a higher power to avoid the stochastic nature of mortality and establish optimal delayed-reperfusion time-points for patients that may otherwise have no viable therapeutic options.

Lack of research on the sequelae of delayed reperfusion is a major barrier preventing its application into clinical trials. Although there is a paucity of information on delayed reperfusion, some basic science researchers have started to investigate novel delayed reperfusion targets. For instance, Mcbride et al. have reported that following permanent MCAO, reperfusion at 3-days, and even 7-days, resulted in improved functional outcome compared to non-perfused animals (39). In summary, studies must continue to carefully evaluate delayed reperfusion beyond conventional time-points, as well as incorporate effective neuroprotective agents to reduce potential side-effects.

Although not exclusively a form of reperfusion injury, HT after reperfusion may be a primary representation and manifestation of the molecular pathways activated by reoxygenation, exposure to renewed systemic pressures, and delivery of pharmaceuticals such as tPA. Studies have tried to develop imaging criteria to identify high-risk patients. For example, Shinoyama et al. have suggested that time to peak (TTP) mapping, a perfusion-based imaging technique, on admission may identify stroke patients and quantify stroke severity (151). The severity of hypoperfusion in stroke correlates with the risk of hemorrhagic transformation (152). Multiple additional risk factors and predictors of severity of hemorrhagic transformation have been identified (153, 154). Not all hemorrhagic transformation as seen on imaging has equivalent clinical ramifications. The European Cooperative Acute Stroke Study (ECASS II) developed criteria to divide hemorrhagic transformation into subtypes, primarily distinguishing between hemorrhagic infarct without mass effect and parenchymal hematoma with mass effect. Upon admission, perfusion imaging sequences, including TTP and mean transit time (MTP), may provide significant prognostic information to guide efforts to reduce HT. Also, with recanalization beyond 6 h from stroke onset, and using CT perfusion, Renu et al. reported significant associations between CT perfusion calculated infarct volume, DWI core infarct volume, cerebral blood volume, cerebral blood flow, clinical outcomes, and rates of hemorrhage (155). Since BBB disruption contributes directly to HT and edema formation, CT contrast material may effectively discriminate regions of increased BBB permeability (156). Taken together, more precise prognostic indicators for HT will develop in parallel with imaging technology and additional research. Advanced imaging techniques show promise in identifying patients with the optimum balance of therapeutic benefit and risk for complications.

In the absences of reperfusion, penumbra is dependent on perfusion to the penumbra from collateral vessels (157). Although collateral circulation shows promise in sustaining the ischemic penumbra, efficacy of leptomeningeal collaterals is variable among stroke patients. Congenital differences result in a variety of atypical primary and secondary collaterals supplied by the Circle of Willis and leptomeningeal vessels (158, 159). Collateral status is also largely dependent on the health of cerebral vasculature. Degree of atherosclerotic disease and vessel elasticity both correlate with the quality of collateral cerebral arteries (160, 161). In a series of patients evaluated by Sharma et al., collateral vessel status was more predictive of outcomes than time from last known well (162). Neuroimaging may therefore be used as a prognostic tool to determine adequate leptomeningeal collateralization and select patients eligible for delayed recanalization. Theorizing that adequate blood pressure is necessary for perfusion of collateral vessels, Hillis et al. reported that optimized blood pressure management improves collateral blood flow, thereby increasing penumbra perfusion and improving functional outcome (163). Taken together, in addition to focusing on reperfusion therapies, researchers should continue to develop complementary strategies to identify, improve, and leverage collateral vessel status to improve stroke outcomes.

Approximately 325,000 ischemic strokes are caused by large vessel occlusion annually in the USA, and only ~20% are treated with recanalization therapy (39). If the recanalization window is extended, and if recanalization efficacy is increased, many of these patients would be eligible for intervention. Stroke care of the future will benefit from improvements at each major step of care—screening, triage, diagnosis, therapy, prognostication, and recovery. Given the rapid progress of non-invasive monitoring and improved predictive models of stroke, patients with high-risk for stroke will likely benefit from non-invasive at-home monitoring to screen for markers of stroke. Alerted by cost effective, non-invasive monitoring, emergency medical service (EMS) teams will immediately be dispatched, shortening the interval between last known well and initial assessment. If stroke symptoms are present, management could be initiated immediately through tele-consultation with a neurologist while en route to a dedicated stroke center. Although not currently deployed, if technological developments continue at pace, screening technologies may be developed that increase the ability of EMS providers to differentiate between ischemic and hemorrhagic events which would open an early treatment window for reperfusion therapy. Regardless of technological developments, upon arrival at a stroke center, dedicated teams of clinicians, technologists, radiologists, and interventionalists will continue to play a pivotal role in stroke treatment. More hospitals will hire and deploy dedicated teams to streamline rapid screening, diagnosis, and treatment of stroke patients. Rapid availability of advanced imaging techniques will increase in the future as evidence accumulates to support the value advanced imaging techniques in screening for hemorrhage, evaluating penumbra status, assessing collateral vessel grade, and dictating deployment of neuroprotective and reperfusion therapies.

We believe that neuroprotective agents in combination with reperfusion will reduce deleterious side-effects associated with ischemia and reperfusion, especially when tailored to select patient subgroups. An increasing fraction of stroke patients will be treated both within the 6 h window and beyond the traditional time window with improved outcomes. Objective evaluation of collateral vessel status will extend the window for intervention in some patients and will likely improve efficacy of delayed recanalization. In patients with poor quality collateral circulation, dedicated therapies will be deployed to sustain the penumbra until recanalization.

## Concluding Remarks

The magnitude of stroke incidence justifies further investigation of interventions with potential for notable effect sizes (164). Even small improvements in the treatment of ischemic stroke will, nonetheless, have profound effects at the level of populations. Balancing the effect size of neuroprotective intervention against the absence of observed side-effects in animal models, neuroprotective agents are promising for translation and have the potential to advance the field of stroke therapy in combination with recent advancements in reperfusion therapy. The future of stroke treatment lies in expansion of the therapeutic window for recanalization therapy in combination with synergistic cocktails of neuroprotective agents to directly interact with the desired tissue in the penumbra to attenuate ischemia-activated deleterious pathways. New developments in recanalization therapy in combination with therapeutics developed through carefully paralleled animal models will allow for novel, intra-arterial deployment of therapeutic agents over a vastly expanded therapeutic time window and with greater likelihood success. We highlight the novel confluence of recent developments to provide real breakthroughs in stroke therapy.

## Author Contributions

The idea and outline of the present review was made by NM, JC, and JZ. This manuscript was drafted by NM. Critical revisions of the manuscript were made by all authors.

## Conflict of Interest

The authors declare that the research was conducted in the absence of any commercial or financial relationships that could be construed as a potential conflict of interest.

## Abbreviations

APC: activated protein C
ATP: adenosine triphosphate
BBB: blood brain barrier
CT: computed tomographic imaging
DWI: diffusion weighted imaging
HT: hemorrhagic transformation
ICAM-1: intercellular adhesion molecule-1
MCAO: middle cerebral artery occlusion
MMP-9: matrix metalloproteinase-9
MRI: magnetic resonance imaging
MTP: mean transient time
ROS: reactive oxygen species
tPA: tissue plasminogen activator
STAIR: stroke therapy academic industry roundtable
TTP: time to peak.
